# Identification of potential maturity indicators for harvesting cacao

**DOI:** 10.1016/j.heliyon.2020.e03416

**Published:** 2020-02-25

**Authors:** Karen E. Rojas, Maria C. García, Ivonne X. Cerón, Ronnal E. Ortiz, Martha P. Tarazona

**Affiliations:** a Process Engineering and Industrial Systems Research Group, Department of Engineering, Universidad Jorge Tadeo Lozano, Bogotá, Colombia; b Corporación Colombiana de Investigación Agropecuaria (AGROSAVIA), Centro de Investigación Tibaitatá, Mosquera, Colombia; c Corporación Colombiana de Investigación Agropecuaria (AGROSAVIA), Centro de Investigación Nataima, Espinal, Colombia

**Keywords:** Food science, Agriculture, Theobroma cacao, CCN51, ICS95, TSH565, Harvesting decision, Developmental stages, Morphological characteristics, Acidity, pH, Total acidity

## Abstract

Cocoa production is a complex process where the conditions of the raw materials decisively impact the final quality of the product. Three universal clones (CCN51, ICS95, and TSH565) from the Department of Huila in Colombia were evaluated to characterize the ripening process of cocoa fruits. Maturity indicators were identified by following the evolution of basic fruit characteristics, including size, weight, seed count, depth and distance between grooves, width and length of the apex, diameter and length of the seed, moisture content, color parameters, fruit firmness, soluble solids content, pH, and acidity. The results indicated that each cocoa clone has a unique set of ripeness parameters: color for ICS95; firmness and weight of the seed for CCN51; and color, morphological characteristics of the apex and grooves, weight, moisture content, pH, and total soluble solids for TSH565. The establishment of reliable, practical, and objective ripeness indicators for each cocoa clone will allow more homogenous cocoa pods to be selected for fermentation, which will ultimately contribute to improved quality and homogeneity of cocoa and its derived products.

## 1 Introduction

Cocoa is a non-climacteric fruit native to Central America and northern parts of South America (Motamayor et al., 2002) from which different products, such as liquor, butter, cocoa powder, chocolate, oils, and extracts, are manufactured. These products are widely used in the food, cosmetic, and pharmaceutical industries due to their organoleptic and bioactive properties (antidiabetic, anticarcinogenic, and antioxidant), which decrease or prevent the development of cardiovascular (Buitrago-Lopez et al., 2011) and skin diseases (Scapagnini et al., 2014).

According to the Food and Agriculture Organization (FAO) of the United Nations, America is the third-largest cocoa bean-producing region in the world, generating an average of 14.9% of the world's production between 1994 and 2016 (FAO, 2018). Within this group, Colombia stood out with 60,535 tons of cocoa produced in 2017; Huila was the fourth-largest contributing Department with ~7.97% of the national participation (FEDECACAO, 2018).

Colombian cocoa is recognized worldwide for its characteristic taste and aroma, which defines it as fine and aromatic cocoa (ICCO, 2015); these factors allow the product to earn higher market prices. However, quality can be affected by the type of clone, its ripeness, and fermentation and drying conditions, among others, resulting in a final product with variable quality and heterogeneous characteristics (Cardona et al., 2016; Sánchez et al., 2007). This is typically due to the use of non-standardized processes to accommodate the diverse and/or heterogeneous raw materials used (including the clone type and ripeness state) and the prevalence of traditional cultural practices in the conditioning, fermenting, and drying processes, leading to inconsistent product quality (Sánchez et al., 2008). According to the Prospective Research Agenda for the Technological Development of the Cocoa-Chocolate Production Chain in Colombia (MADR, 2007; Flórez et al., 2012), the country has minimum development of products with higher added value, low technification, and the absence of standardized processes for cocoa processing and quality control.

The difficulty of standardizing raw material characteristics is due to the clone, ripeness state (Gutiérrez, 2017), edaphoclimatic conditions (Caligiani et al., 2016), and pretreatment of the pod (Afoakwa et al., 2013). Therefore, understanding the effect of these factors on the characteristics of cocoa beans selected to be fermented and dried is essential to obtaining more homogeneous and high-quality cocoa beans, thereby leading to greater homogeneity in the generation of aroma and flavor precursors during cocoa processing—characteristics that are highly sought after by the chocolate industry. Based on its origin, cocoa is classified as Criollo, Forastero, and Trinitario; the latter is the result of a mixture of the first two. Among the best-known universal clones are ICS (Imperial College Selection), TSH (Trinidad Selection Hybrid), and CCN (Castro Naranjal Collection). The first two have their origin in Trinidad, while the third originated in Ecuador. These fruits have similar color characteristics when they mature, reaching colors between red and orange. Among the studies that have been developed to improve cocoa quality are: the use of stainless steel fermenters (Melo et al., 2013), the establishment of non-invasive analytical methods capable of recognizing cocoa varieties (Vargas et al., 2016), and many others that seek to identify metabolic pathways that give rise to flavor and aroma precursors (Voigt et al., 1994; Kratzer et al., 2009; Aprotosoaie et al., 2016; Kongor et al., 2016). However, despite the importance of ripeness state in the final cocoa quality, no indicators of maturity have been reported in the literature that allow for establishing objectively the ripeness state of cocoa fruits and therefore achieving a homogeneous fruit harvest. Standardization of the process would enable the attainment of a product of higher quality and homogeneity. Accordingly, this study seeks to follow the evolution of basic physicochemical parameters during fruit ripening to identify potential maturity indicators for the three clones: CCN51, ICS95, and TSH565.

## 2 Materials and methods

### 2.1 Plant material

Clones with the highest production in the region—namely, CCN51, ICS95, and TSH565—were selected. The cocoa fruits were harvested under four different ripeness stages in the municipalities of Algeciras (2° 31′ 19″ N, 75° 18′ 52″ W) and Garzón (2° 11′ 46″ N, 75° 37′ 45″ W) in the department of Huila, Colombia. The ripeness states (RS) were established based on days since flowering to ensure that all fruits had exceeded the state of physiological maturity. Subsequently, a visual inspection of the fruit color was carried out based on Table 1. Five fruits per clone and ripeness state were harvested, and color parameters were established. Then the fruits were immediately refrigerated (0–4 °C) and transported to the laboratory of the AGROSAVIA research center in the Department of Cundinamarca, for the laboratory analyses described below.

**Table 1:** Definition of the ripeness states (RS) of cocoa fruits according to a visual color inspection.

RS	ICS 95	CCN 51	TSH 565
2	Intense green to deep purple color	Intense purple color	Intense green to purple color
3	Intense purple color with 30% red grooves	Purple to reddish color with 20% yellow grooves	Purple to reddish color with 30% yellow grooves
4	Purple color with 50% red grooves	Reddish color with 30% yellow grooves	Reddish color with 50% yellow grooves
5	Purple color with 80% red grooves	Reddish to yellow color with 60% yellow grooves	Purple to reddish color with 80% yellow grooves

### 2.2 Analysis methods

#### 2.2.1 Physical analysis

##### 2.2.1.1 Fruit shape and size features

The polar and equatorial diameters, the width and length of the apex, and the depth of the grooves as well as the distances between them were measured per fruit using a digital Vernier caliper (Mituyoyo, São Paulo, Brazil) with the results expressed in millimeters.

##### 2.2.1.2 Cocoa bean features

The diameter and length of the cocoa beans were also measured using the digital Vernier caliper, with the results expressed in millimeters.

##### 2.2.1.3 Weight of fruits, pod husks, and cocoa beans

The fruits were weighed using a digital scale (Sartorius, Madrid, Spain). Subsequently, their parts were separated, weighed, and the weight of the pod husks and the cocoa beans was registered in kilograms.

##### 2.2.1.4 Fruit color

Fruit color was established per fruit, in the CIELAB space, using a digital colorimeter (Minolta, Tlalnepantla, Mexico City, Mexico), which provided the coordinates L* (lightness), a* (red to green scale) and b* (yellow to blue scale). With these parameters, the chroma (saturation, intensity, or color purity, $C^{*}=\sqrt{{a^{*}}^{2}+{b^{*}}^{2}}$, °hue angle, the difference between one color and another (arctan(b∗/a∗)) (Sahin and Summu, 2006), and the color index (1000Xa∗/(L∗b∗)) (Vignoni et al., 2006) were calculated. Such measurements were made for three parts of the fruit: the equatorial area, the upper section, and the lower section.

##### 2.2.1.5 Fruit firmness

Fruit firmness was measured using a texturometer (Chatillon Digital DFIS-50, Shanghai, China) in a normal test with a 3.5 mm diameter cylindrical probe and a speed of 60 mm/min. The maximum load reached was expressed in newtons (N). These measurements were made at six points per fruit, two in the upper part, two in the lower part, and two in the middle or equatorial area.

##### 2.2.1.6 Moisture

Moisture values were established using the AOAC 931.0 method. Five cocoa beans per fruit were used; once extracted, they were weighed with a digital scale (Mettler PE 300 digital scale, Ohio, USA), and placed in a Petri dish inside a recirculating air oven (Autonics model TZ4L, Illinois, USA) at 60 °C until a constant weight was reached. The percentage of moisture was calculated by the difference in weight.

#### 2.2.2 Chemical analysis

##### 2.2.2.1 pH, total soluble solids (TSS), and titratable acidity of the pulp

The cocoa bean pulp from each fruit for each clone and every ripeness state was separated and filtered until 20 mL of extract was obtained; this extract was then used to measure the total soluble solids content, pH, and acidity. The total soluble solids content (°Brix) was measured with a digital refractometer (Atago Co. Ltd, Tokyo, Japan). To measure the pH, a digital potentiometer (Mettler Toledo AG, Schwerzenbach, Switzerland) was used, following the AOAC 970.21 method (AOAC, 2016). Finally, to measure the titratable acidity, a 1:5 solution of the extract was prepared and the AOAC method 942.15 (AOAC, 2016) was employed; this result was expressed in grams of citric acid per kilogram of pulp.

### 2.3 Statistical analysis

The data were subject to analysis of variance (ANOVA), complemented with Tukey's multiple comparison test using the GLM procedure (α ≤ 0.05) using SAS software, version 9.4 (SAS Institute Inc., Cary, NC, USA). A unidirectional analysis was also conducted for deeper detail about the behavior of the clones throughout the ripeness process. In order to establish the effect of the two evaluation factors considered, the analysis was carried out discriminating by a single factor—initially by clone, and subsequently by ripeness state. Finally, a two-way analysis was carried out to establish the interaction between clone and ripeness state.

## 3 Results and discussion

### 3.1 Evaluation of three cocoa materials

In a first analysis, an assessment of the results by material (i.e., clones CCN51, TSH565, and ICS95) was carried out without considering the effect of the ripeness state, looking for general parameters that allow differentiation of materials regardless of their ripeness state, since these three clones have remarkably similar physical features that hinder their differentiation. Furthermore, a precise knowledge of what is being fermented is critical to guarantee the quality and homogeneity of the cocoa obtained and its products.

#### 3.1.1 Physical characterization

The results identifying the parameters that differentiate the clones assessed are shown in Table 2. Clone TSH565 showed the largest polar diameter (241 ± 23 mm) among the three clones evaluated (*p* = 0.0163). Clone CCN51 stood out with respect to the diameter of the seeds (12.4 ± 0.9 mm), exceeding the values obtained for clones TSH565 and ICS95 (*p* < 0.0001). Regarding the seed weight, clone CCN51 reported an average value (156 ± 59 g) that was significantly higher than clone ICS95 (*p* < 0.0001); this parameter is directly related to yield since cocoa beans are the raw material used in this agribusiness. As for the length of the seeds clone TSH565 registered the most extended length (24 ± 2 mm), significantly higher (*p* = 0.0212) compared to clone ICS95.

**Table 2:** Parameters with significant differences according to the cocoa materials assessed.

Parameters	Material		
	CCN51	TSH565	ICS95
Polar diameter (mm)	228 ± 16^b^	241 ± 23^a^	222 ± 22^b^
Grain diameter (mm)	12.4 ± 0.9^a^	11.2 ± 0.8^b^	11.5 ± 0.9^b^
Grain weight (g)	156 ± 59^a^	132 ± 40^ab^	118 ± 43^b^
Grain length (mm)	23 ± 1 ^ab^	24 ± 2^a^	23 ± 2^b^
L*	28 ± 8^a^	25 ± 7^b^	24 ± 4^b^
a*	16 ± 8^a^	13 ± 9^b^	11 ± 3^c^
b*	12 ± 9^a^	11 ± 7^ab^	9 ± 5^b^
Chroma, C*	22 ± 10^a^	18 ± 10^b^	14 ± 5^c^
Groove depth (mm)	6 ± 1^a^	6.2 ± 0.8^a^	5 ± 2^b^
Distance between grooves (mm)	6 ± 2^b^	6.8 ± 0.6^a^	5 ± 2^b^
Apex width (mm)	24 ± 4^ab^	27 ± 3^a^	22 ± 7^b^

^a-c^ Equal letters in a row mean that there are no significant differences based on the Tukey-Kramer test (*p* < 0.05).

Regarding color, the parameters L* and a* were higher for CCN51; however, only the chroma registered significant differences (*p* < 0.0001), being more intense for CCN51, followed by TSH565, and finally, the least intense ICS95.

Concerning the morphology represented in the depth and distance between grooves, ICS95 had the smallest groove depth (5 ± 2 mm) (*p* = 0.0002), while TSH565 showed the most distance between them (*p* = 0.0002). Likewise, TSH565 also reported a wider apex compared to ICS95 (*p* = 0.0042). Regarding physicochemical characteristics such as total soluble solids (TSS) and pH, there were no significant differences between the materials (*p* = 0.2812).

Physical parameters are useful when identifying the materials that will be taken to fermentation, as in the country a single material is not fermented; usually, fermentation is carried out with a mixture of clones. Previous data (not shown) indicates that when similar clones are fermented, the cocoa and its products are better in quality and more homogeneous.

#### 3.1.2 Chemical characterization

The chemical characterization, which included total soluble solids, acidity, and pH, did not show significant differences between the materials, so no detailed results are shown. The average value for total soluble solids per material was 20 ± 1 °Brix, 15 ± 1 °Brix, and 20 ± 1 °Brix for CCN51, ICS95, and TSH565, respectively. The pH values for all three clones were similar: 3.65 ± 0.03 for CCN5; 3.67 ± 0.04 for ICS95; and 3.60 ± 0.05 for TSH565. Finally, there were differences in the acidity levels, although these were not significant. Clone CCN51 reported the highest value with 0.54 ± 0.06 g of citric acid/kg of pulp, followed by clone ICS95 with 0.47 ± 0.07 g of citric acid/kg of pulp, and clone TSH565 with 0.42 ± 0.07 g of citric acid/kg of pulp.

### 3.2 Evaluation of the cocoa ripening process

Similar to the earlier analysis by clone, a corresponding analysis based on the ripeness state, independent of the clone, was also carried out. Although the clone type is important in the generation of aromas and flavors, the ripeness state determines characteristics such as soluble solids content, acidity, and pH, among others, which drastically affect the development of the fermentation process and therefore the development of aroma and flavor precursors.

#### 3.2.1 Physical characterization

The ripeness state (RS) data in Table 3 showed the depth of the grooves and the width of the apex to be greater for RS4 and RS5 compared to RS2 (*p* = 0.0209); the grain diameter was smaller (*p* = 0.0102) in RS2 compared to RS5. The highest fruit weight (*p* = 0.0055) was reported in the ripeness states RS3 and RS4. Furthermore, the color parameters a*, b*, and chroma C were significantly higher for RS5 (*p* < 0.0001), which confirmed the direct relationship between the evolution of the ripeness state and fruit color. This relationship was especially evident for the more advanced ripeness states, such as RS5. Finally, an inverse relationship was also observed between the ripeness state and seed moisture, since cocoa beans in ripeness state RS5 had the lowest moisture content (*p* = 0.0143) compared to the ones in RS2 and RS3.

**Table 3:** Physical and chemical characterization of cocoa materials in four ripeness states.

Parameters	Ripeness states			
	2	3	4	5
Groove depth (mm)	5 ± 1^b^	6 ± 1 ^ab^	7 ± 1^a^	6 ± 1^a^
Apex width (mm)	21 ± 5^b^	24 ± 3^ab^	26 ± 4^a^	26 ± 6^a^
Grain diameter (mm)	11 ± 1^b^	12 ± 1 ^ab^	11.9 ± 0.7^ab^	12.2 ± 0.8^a^
Fruit weight (g)	504 ± 153^b^	650 ± 168^a^	673 ± 188^a^	635 ± 206 ^ab^
a*	11 ± 7^b^	11± 8b	13± 6^b^	19± 7a
b*	10 ± 9^b^	9 ± 6^b^	10± 5^b^	14± 8^a^
Fruit Chroma C*	15 ± 11^b^	16 ± 9^b^	17 ± 6^b^	24 ± 8^a^
Grain moisture (g/kg)	765 ± 151^a^	762 ± 64^a^	665 ± 55 ^ab^	604 ± 99^b^
Soluble solids (°Brix)	13 ± 5^c^	16 ± 6^bc^	21 ± 10 ^ab^	22 ± 8^a^
Pulp pH	3.7 ± 0.2^a^	3.7 ± 0.2^ab^	3.6 ± 0.2 ^ab^	3.6 ± 0.2^b^

^a-c^ Equal letters in a row mean that there are no significant differences based on the Tukey-Kramer test (*p* < 0.05).

#### 3.2.2 Chemical characterization

The content of soluble solids and pH showed behavior typical of the ripening processes of any fruit, wherein the first parameter increased while the second decreased to confer better flavor and aroma characteristics to the fruit. Cocoa was not an exception, as its soluble solids content also showed a direct relationship with the ripeness state, reaching the highest content in RS5, followed by RS4, RS3, and RS2 (Table 3). In contrast, the pH showed an inverse relationship with ripeness; however, it only reported significant differences between RS5 and RS2 (*p* = 0.0439). In order to reach specific conclusions by clone type, the ripeness process of each of the three clones evaluated was analyzed, and the results are described below.

### 3.3 Evaluation of physical characteristics during the ripening process of each clone

#### 3.3.1 Morphological and size characteristics

**Figure 1:**
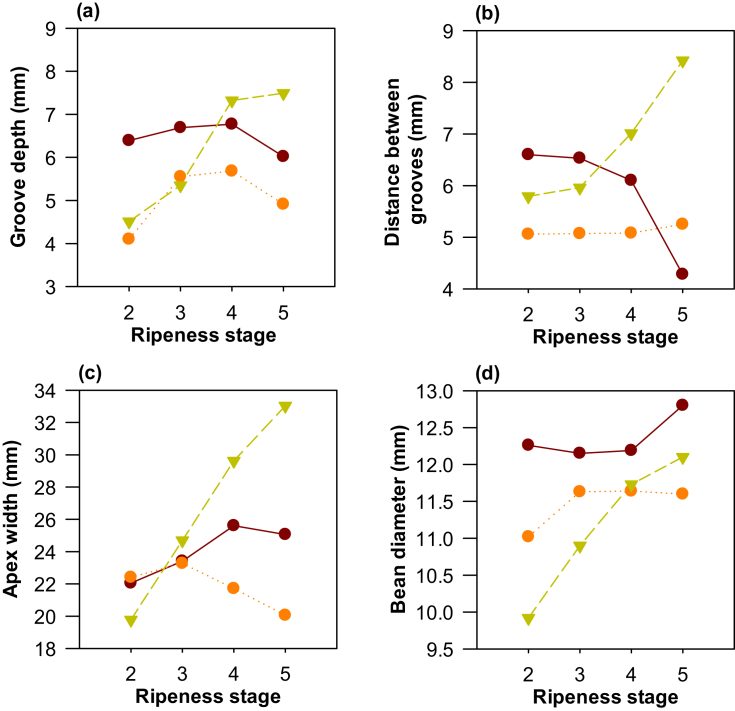
Physical characterization of the cocoa fruits of three clones (CCN51 , ICS95 , and TSH565 ). (a) Groove Depth, (b) distance between grooves, (c) apex width, and (d) bean diameter.

The results for the physical and morphological variables of clone TSH565 indicated increases in the depth of the grooves (Figure 1a), distance between grooves (Figure 1b), apex width (Figure 1c), and seed diameter (Figure 1d), with progressive maturity. However, the distance between the grooves was unaffected by the degree of maturity, by the length of the apex or grain, and the equatorial and polar diameters. The fruit and almond lengths were like those reported for the Brazilian TSH565 cocoa clone at 226 ± 18 mm and 28.3 mm, respectively (Bastos et al., 2018), indicating that the results found in this study were reliable and slightly affected by edaphoclimatic conditions.

Clones CCN51 and ICS95 did not show significant differences in fruit or grain size parameters. Only the distance between grooves was significant for clone CCN51, showing an inverse relationship while maturity progressed (Figure 1b).

The polar diameter results agreed with what was reported for clone CCN51 from the Department of Santander in Colombia (198.12–227.48 mm) (Cubillos et al., 2019), while the equatorial diameter (86.71–97.35 mm) was higher compared to the results reported in that study. According to the results of this study, parameters associated with the size of the fruits cannot be recommended as harvest indicators, since no relationship was found between these and the ripeness state for any of the three clones. This indicates that during the ripening process, fruit growth does not occur; on the contrary, it is more likely that the fruit will begin to lose weight or reduce in size due to dehydration or senescence.

The results obtained show that TSH565 is the clone that presented the highest number of discriminatory factors by ripeness state. These factors also serve as easy evaluation parameters for producers or harvesters, thereby facilitating easy selection of the fruits for harvesting.

#### 3.3.2 Weight of fruit parts and number of seeds

**Figure 2:**
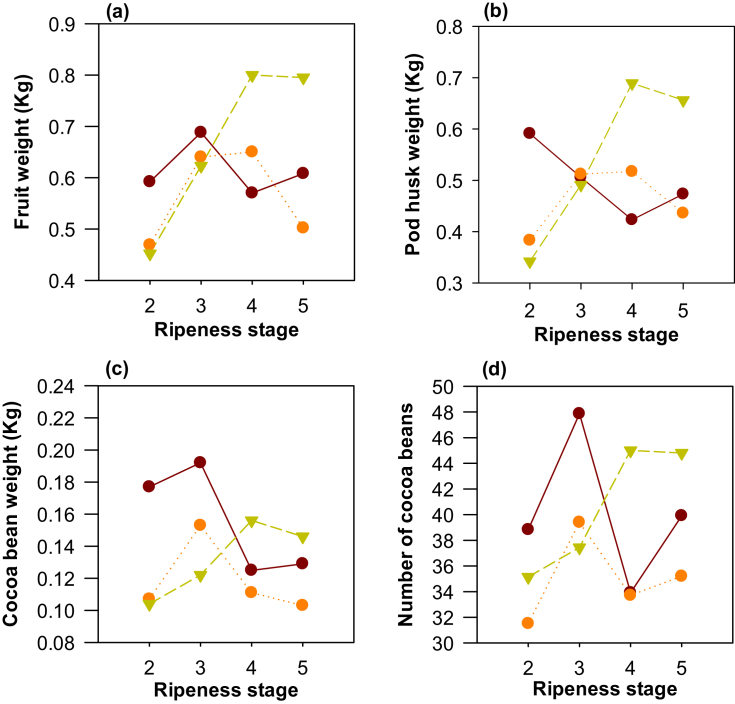
Weight of fruit parts and number of beans in three cocoa clones (CCN51 , ICS95 , and TSH565 ). (a) Fruit weight, (b) pod husk weight, (c) cocoa bean weight, and (d) number of cocoa beans.

The results related to these variables are presented in Figure 2. There was a clear increase in the weight of the fruit with the maturation progress for clone TSH565. Significant differences in fruit weight were found based on the ripeness state (0.5 ± 0.2 kg/fruit in RS1 to 0.8 ± 0.3 kg/fruit in RS4), as shown in Figure 2a. This difference can be attributed to the weight of the pod husk (Figure 2b), which increased with the progress of maturity (0.3 ± 0.1 kg/fruit in RS1 and 0.66 ± 0.07 kg/fruit in RS4), while the weight and number of seeds did not show significant differences. The weight gain of the fruit and the pod husk could be related to filling and maturation processes of the material expressed by length parameters; such measures could jointly describe the ripeness states of clone TSH565.

Fruit and seed weights as well as the number of seeds in RS4 were similar to those found by Bastos et al. (2018), who reported values of 0.705 kg/fruit, 0.136 kg/fruit and 37–52 seeds respectively, for the Brazilian TSH565 cocoa clone, indicating that the RS4 state is the most suitable for fermentation.

**Figure 3:**
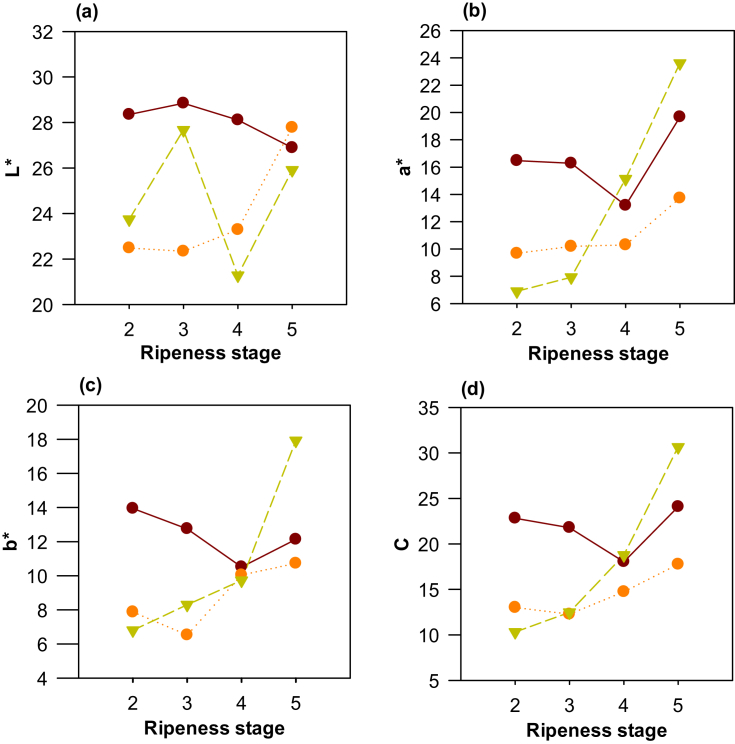
Color parameters of the cocoa and pod husk of three cocoa clones (CCN51 , ICS95 , and TSH565 ). (a) L*, (b) a*, (c) b* and (d) chroma C.

Concerning clone CCN51, significant differences (*p* = 0.0035) in seed weight were found (Figure 3c), decreasing with fruit ripeness (from 0.19 ± 0.05 kg/fruit in RS2 to 0.13 ± 0.05 kg/fruit in RS4). The results regarding seed quantity (40 ± 5 seeds/fruit) were like those reported for the Ecuadorian CCN51 clone (40.44 seeds/fruit with a coefficient of variation of 7.47%) (Vera and Vallejo, 2014).

The weight of the pod husk reported in some clones can become an economic and environmental problem if it is not managed properly. Therefore, research has been carried out to establish the potential of this by-product; several authors have found that compounds such as pectins for pharmaceutical use (Adi-Dako et al., 2018), the fermentation product xylitol (Santana et al., 2018), and skincare gels (Abdul et al., 2016) can be obtained from pod husks. Hence, the large weight of the pod husk can be an advantage if it is used as raw material for other industries that generate products with high added value.

Clone ICS95 did not show significant differences between ripeness states for the parameters evaluated. Therefore, of the parameters associated with the weight of both the fruit and the seeds, fruit weight can be used as a maturity criterion only for clone TSH565. Although in clone CCN51 the weight of the grains was significant, it is not a useful indicator of ripeness in this cocoa clone.

#### 3.3.3 Fruit color

Clones ICS95 and TSH565 presented significant increases in parameters a*, b*, and chroma according to the ripeness state (Figure 3b–d); this represents color development towards red (with positive a*), and yellow (with positive b*) alongside a higher color intensity in advanced ripeness states (Figure 3d). In addition, parameter L* increased for ICS95 (Figure 3a), showing that for this clone the brightness of the bark increases according to the ripeness state. For clone CCN51, there was a significant difference in the values of a* in RS3 (13 ± 7) and RS4 (20 ± 5).

Although these parameters can be used as indicators of the ripeness state for these clones, it is necessary to generate reference patterns or some type of application that allows establishing the intensity (chroma) and the parameters of a* and b* in an objective way to facilitate its use by producers.

None of the clones showed significant differences in the hue angle, with values averaging 35.66–39.95 °hue, or in the color index, with values averaging 55.74–69.83 °hue. These color index values are like those reported for clone CCN51 of Santander, Colombia (40 ± 23 °hue) (Cubillos et al., 2019). The range of colors between deep orange and deep red (Vignoni et al., 2006), as well as the lack of homogeneity in fruit color, requires more precise measurement protocols to reduce or eliminate the noise generated by color variability on the fruit.

#### 3.3.4 Firmness

**Figure 4:**
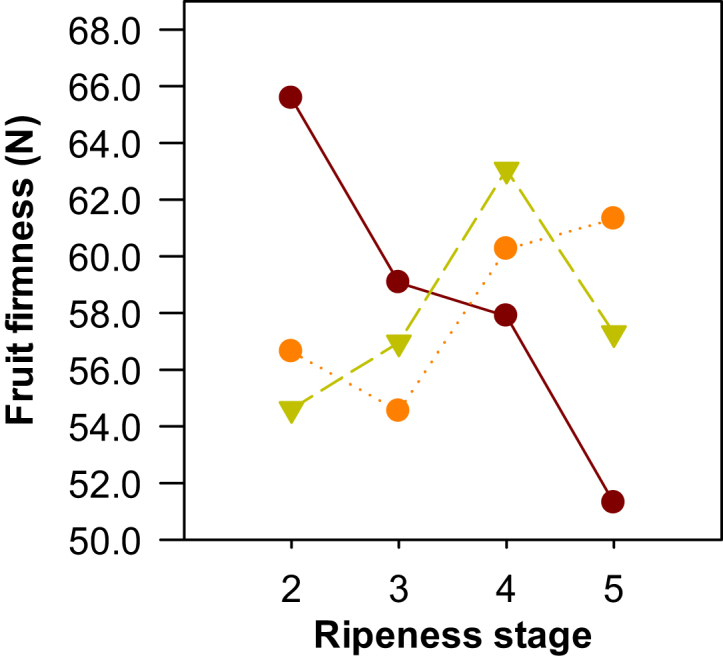
Evolution of fruit firmness during the ripening stages of three cocoa clones (CCN51 , ICS95 , and TSH565 ).

CCN51 was the only clone that showed a significant decrease in fruit firmness (*p* = 0.01) (Figure 4) as the maturity state increased; this was different from what has been reported in a similar study, which found no significant changes in firmness with maturity for clone CCN51 (Cubillos et al., 2019).

This behavior may be related to the high fiber and pectin content of the bark (Vriesmann et al., 2011; Yapo et al., 2013), which can degrade to components that are ethylene-independent and can affect the texture (Pech et al., 2008); this process is more pronounced in climacteric fruits such as papaya, mango, and banana (Torres et al., 2015). Therefore, firmness could be a parameter to establish the ripeness state of clone CCN51.

In contrast, clones ICS95 and TSH565 did not show significant differences in firmness, with values of 58 ± 3 N and 58 ± 4 N, respectively. The non-climacteric behavior of the fruit and the possible low concentration of the independent ethylene compounds that cause softening of the fruit could be the reason for the null behavior of this parameter for clones ICS95 and TSH565. This prevents firmness from being used as a factor that defines ripeness states in these two clones.

#### 3.3.5 Moisture

Only clone TSH565 showed significant differences (*p* = 0.0323) in seed moisture content values for different ripening states (values decreased from 863 ± 41 g of water/kg in RS1 to 533 ± 64 g of water/kg in RS4). Therefore, this could be considered an indicator of ripeness for clone TSH565, although it would be a destructive test. However, it would be easy to apply since it only involves sampling, measuring moisture content, and analyzing the resulting information. Moisture content could thus be a factor that determines the ripeness status for clone TSH565.

For clones CCN51 and ICS95, the moisture measurement values did not change with different ripeness stages, reporting seed moisture contents of 730 ± 7 g of water/kg for clone CCN51 and 670 ± 68 g of water/kg for clone ICS95. Therefore, moisture is not a decisive factor in establishing the ripeness state for clones CCN51 and ICS95. Furthermore, seed moisture results of clone CCN51 are higher compared to the values reported for the Peruvian CCN51 clone (591 ± 7 g of water/kg) (Peláez et al., 2016).

#### 3.3.6 Total soluble solids (TSS), pH, and acidity in pulp

**Figure 5:**
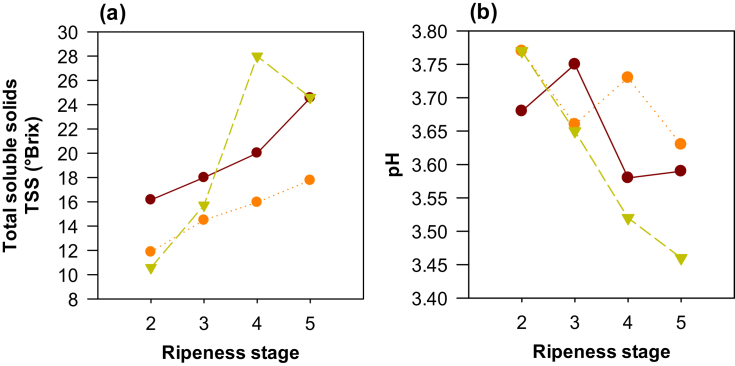
Chemical parameters in the pulp of three cocoa clones (CCN51 , ICS95 , and TSH565 ) throughout their ripeness stages. (a) total soluble solids, (b) pH.

As seen in Figures 5a and 5b, only clone TSH565 showed significant differences in total soluble solids and pH values of the pulp for different ripeness states. The total soluble solids values increased and the pH decreased with increasing maturity. This can be explained by the degradation of starch to soluble sugars and the reduction of organic acids during fruit maturity (Prasad et al., 2018). The pH values for RS4 (3.46 ± 0.23) are similar to those reported by Bastos et al. (2018) for the Brazilian TSH565 clone (3.40 ± 0.03). However, the titratable acidity remained unchanged at 4.1 ± 0.3 g of citric acid/kg of pulp throughout the different ripeness stages. Therefore, soluble solids and pH values can be used to identify the ripeness state of clone TSH565.

These variables are critical for the cocoa fermentation process, controlling its development to a high degree, since they affect the development of yeast and bacteria that participate in the process, as well as the biochemical changes that occur inside the grains and are highly dependent on the pH of the medium. Therefore, given their role in the generation of aromas, flavors, and their precursors, it is essential to select the cocoa clone that has the most favorable pH and TSS characteristics.

For clones CCN51 and ICS95, none of the parameters showed significant differences between the various ripeness states. Total soluble solids depend on genetic factors, as found in this study; they are also affected by environmental conditions, soil composition, water availability, and agronomic practices, all of which can speed up or slow down the conversion of starch to sugar (Cubillos et al., 2019). The results of titratable acidity (4.7 ± 0.1 and 5.4 ± 0.7 g citric acid/kg of pulp for clones ICS95 and CCN51) were lower than the values reported for clone CCN51 in the Department of Santander, Colombia (13.2–17.3 g of citric acid/kg of pulp) (Cubillos et al., 2019). In summary, none of the chemical parameters could be used to identify ripeness states in clones ICS95 and CCN51, although they showed similar results to those reported by Vallejo et al. (2010) for the Ecuadorian clone CCN51 (pH 3.81 and 15 °Brix).

The results allowed us to have greater clarity on the changes that the fruit undergoes throughout the ripening process and once it has passed its multiplication and cell growth stages. Moreover, it also allowed us to observe how the clones behave differently during maturation.

## 4 Conclusions

There are physical differences between clones CCN51, TSH565, and ICS95 that can facilitate their identification. Clone CCN51 has cocoa beans with larger diameters and a higher bean weight compared to clone ICS95, as well as higher color intensity (chroma) compared to the other two clones. Clone TSH565 has a larger polar diameter, a more considerable distance between grooves, and a wider apex compared to the other two clones. Clone ICS95 has a lower groove depth and lower color intensity or saturation compared to the other two clones.

With respect to the degree of ripeness, advanced stages such as RS3 and RS4 have greater groove depth and apex width, greater color intensity, as reflected in the chroma, and higher a* and b* values. Conversely, the moisture content decreases with maturity stages. Regarding the assessed chemical characteristics, the TSS content increased, while the pH decreased with maturity.

The clone analyses allow us to recommend the depth of the grooves, apex width, seed diameter, fruit weight, the values of a*, b*, and chroma, TSS content, pH, and moisture content as maturity indicators for clone TSH565. These parameters increased with maturity except for the last two, which decreased with maturity. For clone CCN51, the distance between grooves, firmness, and the “a*, values are recommended as maturity indicators. For clone ICS95, only the color parameters L*, a*, b*, and chroma, which increased with maturation, can be recommended as maturity indicators.

Results show that the clones assessed evolve differently during maturation, so indicators of maturity must be developed for each of them. Clone TSH565 showed the highest discriminant features, so the homogeneity of harvested fruits for this clone can be improved.

## Declarations

### Author contribution statement

Karen E. Rojas: Performed the experiments; Analyzed and interpreted the data.

Maria C. García: Conceived and designed the experiments; Analyzed and interpreted the data; Contributed reagents, materials, analysis tools or data; Wrote the paper.

Ivonne X. Cerón Performed the experiments; Analyzed and interpreted the data; Contributed reagents, materials, analysis tools or data; Wrote the paper.

Ronnal E. Ortiz: Analyzed and interpreted the data; Contributed reagents, materials, analysis tools or data.

Martha P. Tarazona: Analyzed and interpreted the data; Contributed reagents, materials, analysis tools or data; Wrote the paper.

### Funding statement

This work was supported by Ministerio de Agricultura and Desarrollo Rural (MADR) and Corporación Colombiana de Investigación Agropecuaria (Agrosavia).

### Competing interest statement

The authors declare no conflict of interest.

### Additional information

No additional information is available for this paper.
